# Visualization of cecal tumor by near-infrared laparoscopy and intraoperative colonoscopy

**DOI:** 10.1186/s40792-024-01964-0

**Published:** 2024-07-01

**Authors:** Kaori Watanabe, Hiroki Takahashi, Shuhei Uehara, Akira Kato, Yoshiaki Fujii, Takeshi Yanagita, Takuya Suzuki, Hajime Ushigome, Yuzo Maeda, Ryo Ogawa, Yoichi Matsuo, Akira Mitsui, Shuji Takiguchi

**Affiliations:** Department of Gastroenterological Surgery, Nagoya City University Graduate School of Medical Sciences, Kawasumi 1, Mizuho-cho, Mizuho-ku, Nagoya, 467-8601 Japan

**Keywords:** Cecal tumor, Colonoscopy, Laparoscopic surgery, Near-infrared ray

## Abstract

**Background:**

In laparoscopic colorectal surgery, accurate localization of a tumor is essential for ensuring an adequate ablative margin. Therefore, a new method, near-infrared laparoscopy combined with intraoperative colonoscopy, was developed for visualizing the contour of a cecal tumor from outside of the bowel. The method was used after it was verified on a model that employed a silicone tube.

**Case presentation:**

The patient was a 77-year-old man with a cecal tumor near the appendiceal orifice. Laparoscopy was used to clamp of the terminal ileum, and a colonoscope was then inserted through the anus to the cecum. The laparoscope in the normal light mode could not be used to identify the cecal tumor. However, a laparoscope in the near-infrared ray mode could clearly visualize the contour of the cecal tumor from outside of the bowel, and the tumor could be safely resected by a stapler. The histopathological diagnosis of the resected specimen was adenocarcinoma with an invasion depth of M and a clear negative margin.

**Conclusions:**

This is the first report of the laparoscopic detection of the contour of a cecal tumor from outside the bowel. This technique is useful and safe for contouring tumors in laparoscopic colorectal surgery and can be used in various surgeries that combine endoscopy and laparoscopy.

## Introduction

During laparoscopic-/robotic-assisted resection of colorectal tumors, accurate localization of the tumor is essential for ensuring adequate margins. Inadequate margins may lead to local recurrence [1, 2]. To our best knowledge, there have not been any reports on methods for the accurate contouring of colorectal tumors in laparoscopic surgery.

We previously reported a technique that did not use fluorescent dyes for localizing tumors, but instead combined near-infrared laparoscopy with intraoperative colonoscopy [3]. We used this method to develop a new technique that contours the tumor from outside of the bowel. In this case report, after verifying the technique on a silicon tube model, we found we could use it to provide a clear delineation of the contour of a cecal tumor from outside of the bowel.

## Case presentation

### Presenting history

**Fig. 1:**
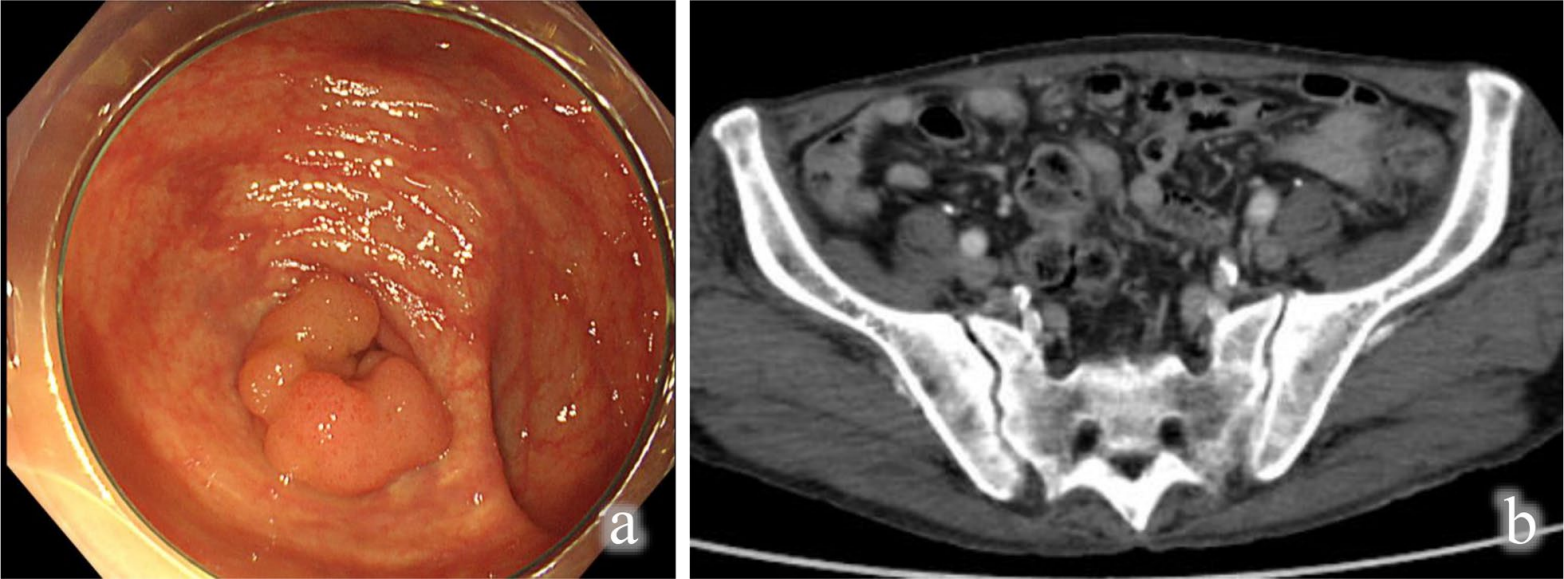
Preoperative examinations. **a** Colonoscopy findings. A 15-mm sessile serrated polyp was identified near the junction of the cecum and appendix; **b** computed tomography (CT) showed no metastases to other organs or lymph nodes

The patient was a 77-year-old man presenting with a positive fecal occult blood test. A colonoscopy revealed a 15-mm sessile serrated polyp near the junction of the cecum and appendix (Fig. 1a). Histopathological analysis classified the polyp as a Group 3 tubular adenoma. An endoscopic submucosal dissection that was considered an appropriate therapeutic intervention for this patient was stopped because of a suspected injury to the muscularis propria. We then decided to perform a surgical resection. Computed tomography was negative for metastases to other organs or lymph nodes (Fig. 1b).

### Preoperative verification of the new technique

**Fig. 2:**
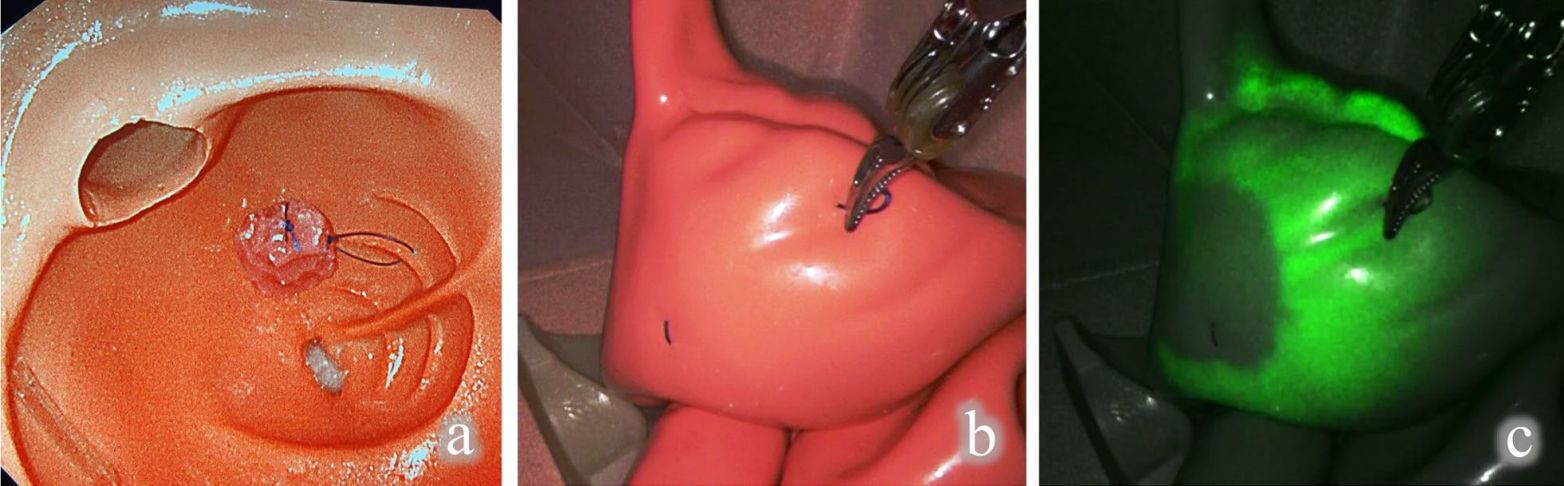
Verification on a silicon model. **a** Observation within the lumen by a colonoscope; **b** normal light observation from outside the bowel by a laparoscope; **c** near-infrared ray observation from outside the bowel by a laparoscope (Da Vinci Firefly mode)

A piece of silicone rubber was used to form a mass that mimicked a colorectal tumor. It was attached within the intestinal lumen of a silicone model that was used for colonoscopy practice. We created a situation similar to that which would be seen in vivo, using a laparoscopic dry box and the silicone model, and confirmed visualization of the mass-like object in the intestinal lumen by a colonoscope (Olympus Corporation, Tokyo, Japan) (Fig. 2a). We used a da Vinci Xi surgical system (Intuitive Surgical Inc., Sunnyvale, CA, USA), to observe the serosal surface by normal light and near-infrared ray. Although mass-like objects attached in the lumen could not be visualized by the laparoscope in the normal light mode (Fig. 2b), when the laparoscope was in the near-infrared ray mode called “Firefly mode”, the mass-like object in the lumen could be clearly contoured from outside the intestinal tract (Fig. 2c).

### Surgical procedure

**Fig. 3:**
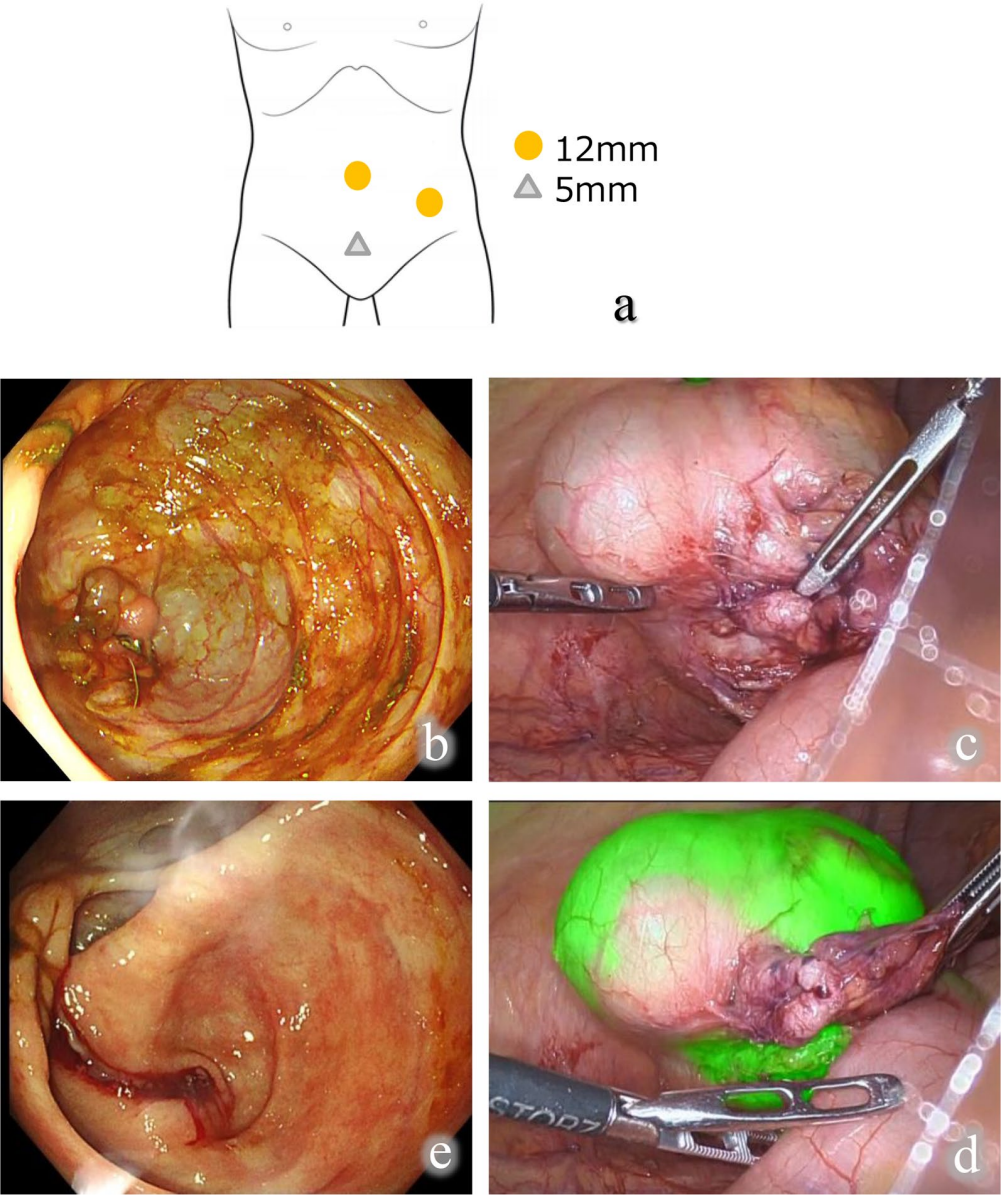
Intraoperative findings. **a** Schematic of port placement; **b** observation within the lumen by a colonoscope; **c** normal light observation from outside the bowel by a laparoscope; **d** near-infrared ray observation from outside the bowel by a laparoscope (Stryker 4K SPY mode); **e** colonoscopy after resection

Three trocars were inserted after the induction of general anesthesia (Fig. 3a). The insufflation of the abdominal cavity to an intraabdominal pressure of 10 mm Hg was then performed. A 1688AIM 4K camera system (Stryker Japan K.K., Tokyo, Japan) was used to enable surgery to mobilize the cecum. After clamping of the terminal ileum by a detachable clamp device, a colonoscope (Olympus Corporation) was inserted through the anus up to cecum to confirm the cecal tumor (Fig. 3b). The laparoscope in the normal light mode did not allow exterior visualization of the location and contour of the cecal tumor (Fig. 3c). However, when the light mode of the laparoscope was changed to near-infrared ray called “SPY mode”, the contour of the cecal tumor could be clearly seen from outside the bowel (Fig. 3d). The cecal tumor was then safely resected by a stapler without injury, while preserving the Bauhin valve (Fig. 3e). The operation time was 1 h and 43 min, the blood loss was 24 ml, and the time to reach the cecum after inserting the colonoscope was 7 min and 30 s.

### Results

**Fig. 4:**
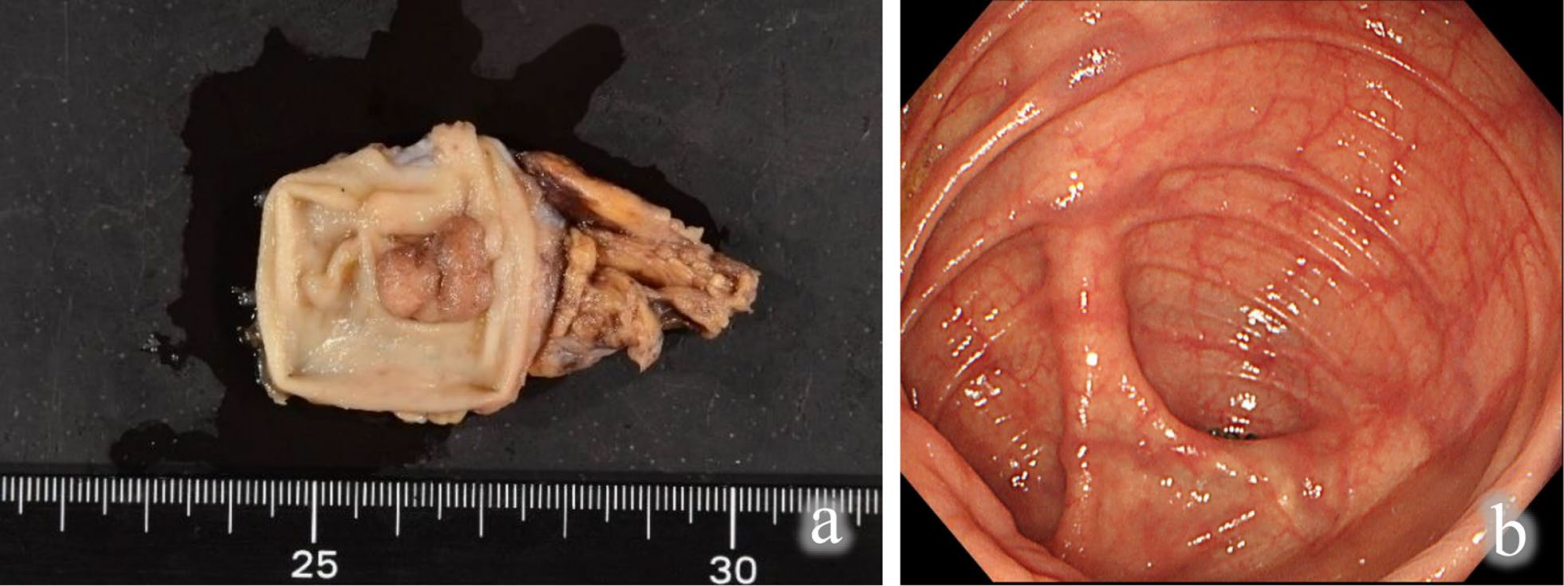
Results. **a** Resected specimen. Histopathological examination showed negative margins; **b** colonoscopy at 1 postoperative year was negative for recurrence

The resected specimen had adequate margins (Fig. 4a). The histopathological diagnosis was well differentiated adenocarcinoma, with an invasion depth of M and negative resection margins. There were no postoperative complications, and a 1-year postoperative colonoscopy was negative for local recurrence (Fig. 4b).

## Discussion

Currently, surgery for colorectal cancer has generally consisted of laparoscopic-assisted and robotic-assisted resection [4, 5]. However, for colorectal tumors, locating the exact position of a colorectal tumor by laparoscopic-/robotic-assisted surgery has been difficult, because the surgeon cannot depend on his/her sense of touch. Inadequate margins or accidental cutting of the tumor may lead to local recurrence and decreased survival times [1, 2]. Thus, the accurate identification of the location of the tumor during laparoscopic colorectal surgery is essential. Endoscopically India ink tattooing or intraoperative colonoscopy have been used to identify the locations of colorectal tumors [6, 7]. However, to our best knowledge, there have been no reports on methods for accurate visualization of the contour of a colorectal tumor during laparoscopic surgery. A more accurate diagnostic method that includes localization has been required. Therefore, we focused on near-infrared ray-guided surgery (NIRGS).

Near-infrared spectroscopy is a diagnostic method that uses a device that can recognize near-infrared rays (700–900 nm) to visualize anatomical structures in the body [8]. Near-infrared rays have very high biological penetration, and NIRGS uses near-infrared spectroscopy. NIRGS is generally used in combination with fluorescent dyes such as indocyanine green (ICG), a water-soluble compound with a molecular weight of 776 Daltons [9]. When ICG is stimulated by near-infrared ray of about 780 nm, it emits fluorescent light at a longer near-infrared wavelength (peak wavelength of 820 nm). This property is used to visualize various anatomical structures such as blood vessels, lymphatic vessels, and bile ducts. It can be used to evaluate blood flow in the gastrointestinal tract and cardiac bypass vessels [10, 11], identify sentinel lymph nodes and the extent of lymph node dissection [12, 13], and identify tumor resection margins [14]. Therefore, it aids in reducing the risk of complications [10, 15, 16]. Devices capable of this diagnostic method are mounted on the da Vinci Xi Surgical System (Intuitive Surgical Inc.), the 1688 AIM 4K camera system (Stryker Japan K.K.), VISERA ELITE II system (Olympus Corporation), PDE (Hamamatsu Photonics K.K., Shizuoka, Japan), and Hyper Eye Medical System (Mizuho Medical Co., Ltd, Nagoya, Japan). Surgery that employs these devices that can recognize the near-infrared wavelengths is referred to as NIRGS. However, most of the existing reports were based on the use of fluorescent dyes.

We previously reported on a useful technique for tumor localization that used near-infrared laparoscopy in combination with intraoperative colonoscopy without the use of fluorescent dyes [3]. In the current report, we further applied this technique to develop a new method for visualizing the contour of colorectal tumors without the use of fluorescent dyes. Since near-infrared ray is reported to penetrate biological tissues up to approximately 5 mm [17], near-infrared ray that penetrates the thin wall of the bowel cannot be visualized exterior to the intestinal tract by normal light, but can be visualized by a camera in near-infrared ray mode. With the laparoscopic camera in this mode, the intraoperative colonoscope illuminates the tumor from inside the bowel, making it possible to visualize not only the location of the tumor by the laparoscope, but also its contour from outside the bowel. The resection line can thus be determined, and preservation of function can be ensured without leaving residual tumor or needing to enlarge the extent of resection. Another advantage of this technique is that it avoids the risk of an allergic reaction caused by fluorescent dyes such as ICG, which contains iodine and is contraindicated in patients with iodine hypersensitivity [18].

This NIRGS technique has some limitations. First, we used the 1688 AIM 4K camera system, the da Vinci Xi surgical system, and an Olympus colonoscope, but we have not used other near-infrared devices or colonoscopes, and must find out if other devices can be used. Second, it is necessary to perform a colonoscopy during the procedure, which requires a person with endoscopic skills and additional operation time to insert the colonoscope. In addition, while insufflation with the colonoscope can make laparoscopic manipulation difficult, the increased mobility of the colon under pneumoperitoneum also complicates endoscope insertion. Therefore, not only should the terminal ileum be clamped, but also, after mobilizing the cecum, it is better to temporarily release the pneumoperitoneum before inserting the endoscope. The difficulty of laparoscopic manipulation can be avoided by allowing the endoscope to reach the cecum with minimal insufflation and then reestablishing pneumoperitoneum. Third, the resection is limited to tumors that do not require lymph node dissection, specifically benign tumors, endoscopically challenging mucosal cancers, and submucosal tumors. However, depending on the thickness of the bowel and the amount and location of attached fat, visualizing the tumor may be difficult. And last, the risk of local recurrence has not been evaluated.

The NIRGS technique presented in this report enables visualization of the contour of the colorectal tumor without the use of fluorescent dyes, and safe resection with adequate margins while preserving function, such as maintaining the Bauhin valve and avoiding intestinal stenosis. In addition, even in cases where the appendix is absent as a landmark following appendectomy, the tumor's location and extent can be accurately identified. Although further validation is required, we believe that this technique can be applied to laparoscopic and endoscopic cooperative surgery (LECS) for submucosal tumors of the stomach and duodenum. Although this technique should be carefully considered for use in selected cases, in the future, it may be applicable to a variety of combined laparoscopic and endoscopic surgeries.

## Conclusions

The NIRGS technique is safe and useful for the detection of tumor contour during laparoscopic colorectal surgery. We think that it will be applicable to various surgical procedures that combine endoscopic with laparoscopic surgery.

## Data Availability

The data sets used and/or analyzed during the current study are available from the corresponding author on reasonable request.

## Abbreviations

CT: Computed tomography
ICG: Indocyanine green
LECS: Laparoscopic and endoscopic cooperative surgery
NIRGS: Near-infrared ray-guided surgery
